# Propolis Extract Reduces Doxorubucin‐Induced Brain Damage by Regulating Inflammation, ER Stress, Oxidative Stress, and Apoptosis

**DOI:** 10.1002/fsn3.70194

**Published:** 2025-04-22

**Authors:** Volkan Gelen, Mehmet Başeğmez, İnan Dursun, Irfan Çinar, Adem Kara

**Affiliations:** ^1^ Department of Physiology, Faculty of Veterinary Medicine Kafkas University Kars Türkiye; ^2^ Acıpayam Vocational High School, Department of Veterinary, Laboratory and Veterinary Health Program Pamukkale University Denizli Türkiye; ^3^ Center of Research and Application Center Bingöl University Bingöl Türkiye; ^4^ Department of Crop and Animal Production, Vocational School of Food, Agriculture and Livestock Bingöl University Bingöl Türkiye; ^5^ Department of Pharmacology and Toxicolog, Faculty of Medicine Kastamonu University Kastamonu Türkiye; ^6^ Department of Genetics, Faculty of Science Erzurum Technical University Erzurum Türkiye

**Keywords:** apoptosis, DOX, inflammation, oxidative stress, propolis extract, UHPLC‐Orbitrap‐HRMS

## Abstract

Doxorubicin (DOX) is the most widely used chemotherapeutic agent to treat various tumors. DOX treatment can damage many organs, including the brain, by causing oxidative stress. Several antioxidant substances can lessen the effects of DOX or make antioxidant defense systems work faster. Propolis (PROP) is a powerful agent with various healing effects, including antioxidant, antiproliferative, and anti‐inflammatory. The point of this study is to look at the histopathological changes, apoptosis, and antioxidant effects of DOX on brain damage in rats. To find out what kinds of phytochemicals were in PROP from the Karlıova region of Bingöl province, ultra‐high‐performance liquid chromatography (UHPLC‐Orbitrap‐HRMS) was used. Then, we made an ethanol extract of it. A total of 28 healthy male Wistar albino rats, each 12 weeks old and weighing between 220 and 250 g, were included in the study. Rats were divided into four groups: control, PROP, DOX, and PROP+DOX. We applied the relevant treatments to the determined groups. Following the application, we decapitated the rats under the appropriate conditions and collected blood and brain tissue samples. We examined oxidative stress parameters in blood samples and used brain tissue samples for histopathological, biochemical, and molecular analyses. We determined DOX levels in the brain tissue samples using UHPLC‐Orbitrap‐HRMS. The findings obtained showed that the PROP extract improved DOX‐induced brain tissue damage. In addition, PROP extract attenuated DOX‐induced brain tissue inflammation, ER stress, apoptosis, and oxidative stress.

## Graphical abstract

PROP demonstrated protective effects against DOX‐induced oxidative stress, inflammation, and apoptosis in brain tissue by reducing MDA levels while increasing GSH, SOD, and CAT activities. It also attenuated DOX‐induced increases in inflammatory and apoptotic markers such as TNF‐α, IL‐1β, IL‐6, NF‐kB, iNOS, and CASP‐3 while restoring PI3K, mTOR, AKT activity, and BCL2/BAX expression. Histopathological and molecular analyses confirmed that PROP alleviated neuronal degeneration and modulated key signaling pathways, highlighting its neuroprotective potential.

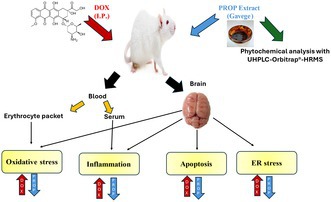

## 1 Introduction

Doxorubicin (DOX) is an anthracycline group antineoplastic agent used in various cancer treatments. Oxidative stress in multiple tissues limits the therapeutic use of the drug in cancer treatment. Free radicals, membrane lipid peroxidation, apoptosis, and inflammation have all been linked to DOX‐induced neurotoxicity (Cagel et al. 2017). The chemical structure of DOX increases oxidative stress and causes cell damage by allowing the formation of free radicals (Alavi and Varma 2020). Under normal conditions, reactive oxygen species produced by aerobic metabolism are constantly inhibited. The antioxidant defense systems in the organism carry out this work, preventing any pathological event from occurring. The organism is not hurt by free radicals as long as the balance between the rate of formation and the strength of the defense systems is not upset. When this balance is disrupted against the antioxidant systems, potential damage occurs, which is called ‘oxidative stress’ (Alavi and Varma 2020; Lesniak et al. 2005; Gu et al. 2021). DOX is one of the causes of the imbalance between free oxygen radicals and antioxidants (Vyas et al. 2020). Tissue damage is seen as protein oxidation and lipid peroxidation in the tissue (Vyas et al. 2020). This is because the oxidant and antioxidant systems are not working together properly. Free radicals and antioxidant enzymes are thought to play a part in DOX‐induced toxicity. This has led to plans to study antioxidant treatments (Sannasimuthu et al. 2019). Some antioxidative agents are used to reduce the possible side effects of anticancer drugs and the oxidative stress that may occur. Various studies (El‐Agamy et al. 2019) have reported the effects of some antioxidants on DOX‐induced damage. One of the most important antioxidant compounds is PROP extract, which contains multiple components. In the past few years, it has become more popular and important to use natural PROP extract to treat and prevent the side effects of chemotherapy (Morean et al. 2015). For this reason, researchers have started to search for natural substances with proven biological effects along with the tendency to use natural substances in this regard. PROP, a natural product that is gaining increasing importance today, is a sticky, dark‐colored resinous substance that honey bees collect from living parts of plants by adding salivary enzymes to them, mixing them with wax, and using them in the hive for various purposes. PROP is a popular medicine in folk medicine, apitherapy, cosmetics, and the pharmaceutical industry. It is used for many things because it has biological activities like anti‐inflammatory, antitumor, anti‐ulcerative, and immunostimulant properties, as well as antibacterial, antiviral, and antifungal properties (Zulhendri et al. 2022; Valverde et al. 2023). The point of this study is to look at how PROP, a naturally occurring substance that has many known biological effects and is easy to get, can protect the brains of rats that have been damaged by DOX. We will do this by using biochemical, molecular, and histopathological findings.

## 2 Materials and Methods

### 2.1 Propolis Extraction

Samples of PROP were purchased fresh directly from beekeepers in the Karlıova region of Bingöl province and stored at −80°C until extraction. PROP ethanol extract preparation involves freezing at −80°C to harden it, followed by grinding it into a powder using a laboratory mill. Subsequently, 500 g of PROP are placed in a 2.5 L amber glass bottle, to which 2 L of HPLC‐grade ethanol is added. After 30 min of ultrasonic treatment and stirring at 500 rpm with a magnetic stir bar at room temperature in the dark for 7 days, the solution is filtered through coarse filter paper and then through a blue band filter paper using a vacuum filtration system. We evaporate the resulting filtrate in a rotary evaporator at 40°C under a vacuum. We store the obtained PROP extract in amber glass containers at 4°C in the dark until use.

### 2.2 Experimental Protocol

#### 2.2.1 Animals and Experimental Model

The study was conducted under the authorization of the Pamukkale University Animal Experiments Local Ethics Committee, with reference number PAUHDEK‐2023/33. All animals were treated according to the Use of Animals and ARRIVE guidelines. A total of 28 healthy male Wistar albino rats, each 12 weeks old and weighing between 220 and 250 g, were included in the study. During the 10‐day study period, rats were housed in a 50%–55% humidity, 22°C ± 1°C temperature, and 12‐h light/dark environment under veterinary control. Rats in Group I (control group) were administered 0.5 mL of ethanol orally. Rats in Group II were administered PROP extract by gastric gavage at a dose of 200 mg/kg/day. Group III rats received DOX intraperitoneally at a dosage of 2 mg/kg, administered five times every other day. DOX was given to Group IV rats intraperitoneally five times every other day at a dose of 2 and 200 mg/kg of PROP extract was given by gastric gavage for 10 days (Table 1).

**TABLE 1:** Groups and applications.

Group name	Application
Control (*n* = 7)	Rats in this group were administered 1 mL of 5% ethanol by gavage for 10 days
PROP (*n* = 7)	The rats in this group were given PROP extract at a dose of 200 mg/kg by gavage for 10 days
DOX (*n* = 7)	DOX was administered at a dose of 2 mg/kg I.P. 5 times every other day
DOX + PROP (*n* = 7)	Rats in this group were administered PROP extract at a dose of 200 mg/kg via gavage for 10 days, as well as DOX at a dose of 2 mg/kg I.P. 5 times every second day

#### 2.2.2 Collection of Blood and Tissue Samples

We took blood samples from the abdominal aorta of rats that had been asleep for 12 h on xylazine (0.01 mg/g per body weight) and ketamine (0.04 mg/g per body weight). Blood samples from the abdominal aorta were collected in anticoagulated EDTA tubes. Following the collection of blood samples, we sacrificed the rats and extracted brain tissue samples for biochemical, histopathological, and molecular analyses. Brain tissue samples were stored at −80°C until the day of analysis.

#### 2.2.3 Preparation of Erythrocytes

We centrifuged the tubes at 3000 rpm for 15 min at 4°C, approximately 1 h after blood collection. We transferred the obtained plasma samples into Eppendorf tubes and stored them at −20°C until analysis. Phosphate buffer solution (PBS) (pH 7.4) was added to the red blood cell packet remaining at the bottom of the EDTA tube. Then, the tubes with PBS were centrifuged at 3500 rpm for 15 min at 4°C. Washing with a PBS solution and centrifugation were repeated three times. PBS solution was then added to the erythrocyte packs in a 1:1 volume ratio. The packs were then kept at −20°C until they were analyzed. Whole blood was used for MDA and GSH levels, and erythrocyte homogenate samples were used for SOD and CAT activity measurements.

#### 2.2.4 Biochemical Analysis

##### 2.2.4.1 Analysis of Oxidative Stress Parameters

The amount of malondialdehyde (MDA), which is a sign of oxidative damage, was measured in whole blood using the method of Draper and Hadley (1990) and given as nmol/mL. The amount of glutathione (GSH) in whole blood was found using spectrophotometry, as described by Beutler et al. (1963). The results were given as nmol/mL for whole blood. Superoxide dismutase (SOD) and catalase (CAT) antioxidant enzyme activities in erythrocyte homogenate were measured according to the methods of Sun et al. (1988) and Luck (1955) and expressed as U/gHb and k/gHb, respectively. The brain tissues were thoroughly washed with cold 0.9% sodium chloride (NaCl) solution. Following this, they were homogenized in 0.1 M phosphate buffer (pH = 7.4) at a ratio of 1:40 (w/v). The resulting homogenates were then centrifuged at 5000 rpm for 15 min, according to the method described by Acaroz et al. Following this, the levels of malondialdehyde (MDA), glutathione (GSH), superoxide dismutase (SOD), and catalase (CAT) were assessed in the supernatants obtained from the centrifuged brain tissue samples.

##### 2.2.4.2 Measurement of PI3K/Akt/mTOR Parameters in Brain Tissue Samples by ELISA Method

We took samples from brain tissues stored at −80°C and weighed them. Tissue samples were homogenized by mixing with PBS. After homogenization, they were centrifuged in a refrigerated centrifuge at 3000 rpm for 20 min. The supernatants obtained were transferred to a separate tube. Samples taken from the prepared supernatants were measured according to the procedure in the commercial ELISA kit (PI3K (201‐11‐0439 Sunred), PKB (201‐11‐0202, Sunred), mTOR (201‐11‐4241, Sunred)), and the results were calculated.

#### 2.2.5 Gene Expression Analysis

We used the Real‐Time PCR method to compare the levels of TNF‐α, IL‐1β, IL‐6, NF‐kB, nNOS, iNOS, Caspase‐3, and BCL2/BAX mRNA in the brain tissues from the different groups.

##### 2.2.5.1 Real‐Time PCR Analysis

###### 2.2.5.1.1 Quantitative Determination of mRNA Expression by Real‐Time PCR

Tissue samples (25–30 μg) were homogenized in a Tissuelyser II (Qiagen) device with liquid nitrogen. RNA extraction will continue in the QIAcube RNA isolation device as the manufacturer recommends (Qiagen, RNeasy Mini Kit, Cat no./ID. 74,104). Total RNA extraction and cDNA synthesis (Applied Biosystems, High‐Capacity cDNA Reverse Transcription Kit, Cat. No. 4368814) were performed according to the methods described in our previous studies [24]. According to the manufacturer's instructions, TNF‐α, IL‐1β, IL‐6, NF‐kB, nNOS, iNOS, Caspase‐3, BCL2/BAX, and β‐actin mRNA expression as a housekeeping gene were analyzed. It was used by synthesizing the gene identified through literature scanning and gene bank control. In addition, the primers used in the study are given in Table 2. Compared with the control group, all data were expressed as fold change in expression compared with the cell groups using the 2^−ΔΔ^Ct method.

**TABLE 2:** Rat primers used for RT‐PCR.

Target gene		Primer sequence: 5′‐3′
TNF‐α	F	CCAGGAGAAAGTCAGCCTCCT
	R	TCATACCAGGGCTTGAGCTCA
IL‐6	F	TCCTACCCCAACTTCCAATGCTC
	R	TTGGATGGTCTTGGTCCTTAGCC
IL‐1β	F	CACCTCTCAAGCAGAGCACAG
	R	GGGTTCCATGGTGAAGTCAAC
NFkB	F	ATCATCAACATGAGAAACGATCTGTA
	R	CAG CGG TCC AGA AGA CTC AG
nNOS	F	CGACCAATACTACTCATCCA
	R	CTCCTTGTTCACCTCCTC
iNOS	F	CACCACCCTCCTTGTTCAAC
	R	CAATCCACAACTCGCTCCAA
BAX	F	ACCAAGAAGCTGAGCGAGTG
	R	CCAGTTGAAGTTGCCGTCTG
BCL‐2	F	CTGGTGGACAACATCGCTCT
	R	GCATGCTGGGGCCATATAGT
Caspase‐3	F	GGAGCTTGGAACGCGAAGAA
	R	ACACAAGCCCATTTCAGGGT
β‐Actin	F	TGTTACAGGAAGTCCCTTGCC
	R	AATGCTATCACCTCCCCTGTG

#### 2.2.6 Western Blot Analysis

We wanted to find out how much reticulum stress, apoptosis, and inflammatory marker protein were present in the brain tissue samples from the experimental groups. To prepare the brain tissue samples for western blot analysis, they were kept at −80°C in a deep freezer. The brain tissue samples were weighed and then crushed in nitrogen gas. They were then used for radioimmunoprecipitation with RIPA buffer (Ecotech Bio, Turkey) that had been strengthened with protease and phosphatase inhibitors. Next, we homogenized the samples using a tissue disruptor (Qiagen, USA) for 20 s at 30 Hz. It establishes the relative levels of ATF6, IRE1, and TLR4 protein expression. We established the overall protein content in the brain tissue. After 10% SDS‐PAGE was used to separate the proteins, 30 μg of protein was added to the PVDF membrane. Membranes were first blocked with 5% bovine serum albumin for 90 min at room temperature. We then left the membranes overnight at 4°C to treat them with the corresponding primary antibodies. PVDF membranes were treated with primary antibodies for 90 min at room temperature. After that, they underwent a TBST wash and were left to incubate with a secondary antibody linked to horseradish peroxidase for an additional 90 min. Protein bands were then identified and examined using the enhanced chemiluminescence reagent Western ECL substrate (Thermo, 3405), and images were captured (Image Lab Software, Bio‐Rad, Hercules, CA, USA). β‐actin was used for the normalization of protein expression levels. For this purpose, the normalization process was performed by dividing the intensity of the target protein by the intensity of β‐actin.

#### 2.2.7 Histopathological Evaluation

The brain tissues of rats in all experimental groups were removed at the end of the experiment and were transferred to 10% neutral buffered formalin solution and fixed for 72 h. Brain tissue samples were dried out by running them through a series of increasing alcohol grades (70%, 80%, 96%, and 100%). The tissue was then cleaned with xylene and paraffin infiltrated to make tissue blocks. Then, a rotary microtome (Leica RM2125 RTS) was used to cut brain tissue blocks into 5 μm thick slices that were then put on slides. We stained the slides using Crossman's Modified Mallory's Triple Staining method for histopathological evaluations. Finally, the preparations were examined and photographed at 200x magnification using a light microscope (Zeiss AXIO Scope.A1) with a modified imaging system. Neuropathological damage in the cerebral cortex was reinterpreted using a scoring system from 0 to 4: 0 meant there was no change, 1 meant less than 10% of the affected area, 2 meant 20%–30% of the affected area, 3 meant 40%–60% of the affected area, and 4 meant more than 60% of the affected area (Ibrahim Fouad and Ahmed 2021).

#### 2.2.8 Analysis of DOX in Brain Tissues and Phytochemical Content Analysis in PROP Extract Using Ultra‐High‐Performance Liquid Chromatography‐Orbitrap‐High‐Resolution Mass Spectrometry (UHPLC‐Orbitrap‐HRMS)

About 1 g of brain tissue samples was taken and put into 2 mL locked Eppendorf tubes so they could be prepared for DOX analysis. To these samples, 500 μL of pH 7.4 phosphate‐buffered saline (PBS) and 500 μL of methanol were added to ensure homogenization. This process was carried out for 15 min using steel beads in the Qiagen Tissue Lyser II device. The homogenized tissue samples were centrifuged at 15,000 rpm for 15 min at 4°C, and the resulting supernatant was stored for analysis. The stock PROP solution was diluted in a solution of 50% methanol and 50%, 0.5% acetic acid in water to adjust the concentration to 1 mg/mL. Before analysis, all samples were passed through a 0.22 μm pore size and 25 mm diameter PTFE (MARZ Shuller) syringe filter using a 10 mL syringe and transferred to 1.5 mL capped vials.

The analysis of DOX in brain tissues and the phytochemical content analysis of PROP extract were carried out using Thermo Fisher Scientific's Exactive Plus Orbitrap mass spectrometer. Chromatographic separation was achieved with the DIONEX UltiMate 3000 RS pump, DIONEX UltiMate 3000 RS autosampler, and DIONEX UltiMate 3000 RS column oven. A MerckPurosper STAR RP‐18 endcapped Hibar HR model UHPLC column (100 mm × 2.1 mm, 3 μm) was used, with the column oven temperature set at 30°C and a mobile phase flow rate of 0.3 mL/min. Mobile phase A consisted of 0.5% acetic acid in water, while mobile phase B was LC–MS grade methanol. The gradient elution was set as follows: 0–2 min: 100% A‐0% B; 2–15.5 min: 0% A‐98% B (ramp); 15.5–15.9 min: 98% B; 16–16.1 min: 100% A‐0% B; 16.1–19 min: 100% A‐0% B. The total method runtime was 20 min, with an injection volume of 20 μL. Mass detection was performed using the Exactive Plus mass spectrometer manufactured by Thermo Fisher Scientific Inc. (Waltham, MA, USA). Ionization was conducted with a heated electrospray ionization (HESI‐II) probe. The HRMS operated in negative electrospray ionization (‐ESI) mode with a mass scan range of 100 to 800 m/z, a negative mode spray voltage of 3.5 kV, a sheath gas flow rate of 35 units, an auxiliary gas flow rate of 7 units, a spare gas flow rate of 0 units, a capillary temperature of 350°C, an auxiliary gas temperature of 350°C, an S‐lens RF level of 50, and a maximum injection time (IT) set to 2 ms. Mass spectra were acquired using two distinct acquisition modes: (1) Full MS mode without fragmentation, with the high‐energy collisional dissociation (HCD) cell closed, and (2) All Ion Fragmentation (AIF) mode with MS/MS fragmentation and HCD open (collision energy = 25 eV). Automatic Gain Control (AGC) was set to 3 × 10^6^. The resolution was 17,500 for both Full MS and AIF modes (Dursun et al. 2025).

### 2.3 Statistical Analysis

SPSS 27.0 (SPSS Inc., Chicago, IL, USA) was used for statistical analysis. GraphPad Prism 9.05 (GraphPad Software, San Diego, CA, USA) was used for graphical presentations. All results are expressed as the mean ± standard error of the mean (SEM). The normality of the data was found to be normally distributed by the Shapiro–Wilk test. In addition, the homogeneity of the data was demonstrated with the homogeneous variance test. To compare multiple groups, the one‐way ANOVA and Duncan test were utilized. The statistical significance level was determined as *p* < 0.05.

## 3 Results

### 3.1 Effects of PROP on Oxidative Stress Parameters in DOX‐Induced Brain Tissue

DOX (DOX) significantly raised MDA levels in whole blood (Table 3) compared to the control group (*p* < 0.05). PROP efficiently decreased the levels of MDA in the DOX + PROP groups (*p* < 0.05). DOX significantly decreased GSH levels compared to the control group (*p* < 0.05). PROP significantly raised GSH levels in the DOX + PROP group (*p* < 0.05). The level of SOD was considerably lower in the group administered DOX compared to the other groups in the study (*p* < 0.05). PROP supplementation significantly increased SOD levels in the PROP group compared to the other study groups (*p* < 0.05). DOX significantly reduced CAT levels compared to the control group and other study groups (*p* < 0.05). PROP significantly increased CAT enzyme activity in the DOX + PROP group (*p* < 0.05). Doxorubicin (DOX) significantly raised MDA levels in brain tissues (Table 4) compared to the control group (*p* < 0.05). Propolis (PROP) significantly decreased the levels of MDA in the DOX + PROP groups (*p* < 0.05). DOX considerably decreased GSH levels compared to the control group (*p* < 0.05). PROP significantly raised GSH levels in the DOX + PROP group (*p* < 0.05). DOX efficiently decreased SOD levels compared to the control group (*p* < 0.05). PROP increased SOD levels, but this did not reach statistical significance (*p* > 0.05). DOX substantially reduced CAT levels compared to the control group and other study groups (*p* < 0.05). PROP significantly increased CAT enzyme activity in the DOX + PROP group (*p* < 0.05).

**TABLE 3:** Effects of DOX and PROP on malondialdehyde and glutathione levels in the whole blood and superoxide dismutase and catalase activities in the erythrocyte homogenate of rats.

Groups	MDA (nmol/mL)	GSH (nmol/mL)	SOD (U/gHb)	CAT (k/gHb)
Control	2.27 ± 0.36^bc^	33.65 ± 1.53^b^	18.80 ± 0.68^b^	17.46 ± 0.45^a^
PROP	2.05 ± 0.20^c^	43.50 ± 3.07^a^	29.31 ± 1.02^a^	19.30 ± 0.74^a^
DOX	4.56 ± 0.45^a^	24.21 ± 2.06^c^	13.14 ± 0.79^c^	11.87 ± 0.74^c^
DOX + PROP	3.14 ± 0.30^b^	35.20 ± 2.10^b^	16.73 ± 1.22^b^	15.13 ± 0.98^b^

*Note:* The values were expressed as means ± SEM (*n*: 7). ^a,b,c^Different letters in the same column represent statistically significant differences (*p* < 0.05).

Abbreviations: CAT, catalase; GSH, glutathione; MDA, malondialdehyde; SOD, superoxide dismutase.

**TABLE 4:** Effects of doxorubicin and propolis on malondialdehyde (MDA) and glutathione (GSH), superoxide dismutase (SOD), and catalase (CAT) levels in brain tissue of rats.

Groups	MDA (nmol/g tissue)	GSH (nmol/g tissue)	SOD (U/μg protein)	CAT (U/μg protein)
Control	0.27 ± 0.02^bc^	18.13 ± 0.69^b^	10.47 ± 1.72^ab^	2.23 ± 0.14^ab^
PROP	0.22 ± 0.03^c^	23.64 ± 0.89^a^	11.02 ± 1.86^a^	2.47 ± 0.06^a^
DOX	0.47 ± 0.05^a^	12.38 ± 0.37^c^	7.39 ± 1.57^c^	1.81 ± 0.03^c^
DOX + PROP	0.38 ± 0.04^b^	16.62 ± 0.45^b^	8.46 ± 1.65^bc^	2.15 ± 0.06^bc^

*Note:* The values were expressed as means ± SEM (*n*: 7). ^a,b,c^Different letters in the same column represent statistically significant differences (*p* < 0.05).

Abbreviations: CAT, catalase; GSH, glutathione; MDA, malondialdehyde; SOD, superoxide dismutase.

### 3.2 Effects of PROP on PI3K, mTOR, and Akt Activities in DOX‐Induced Brain Tissue

**FIGURE 1:**
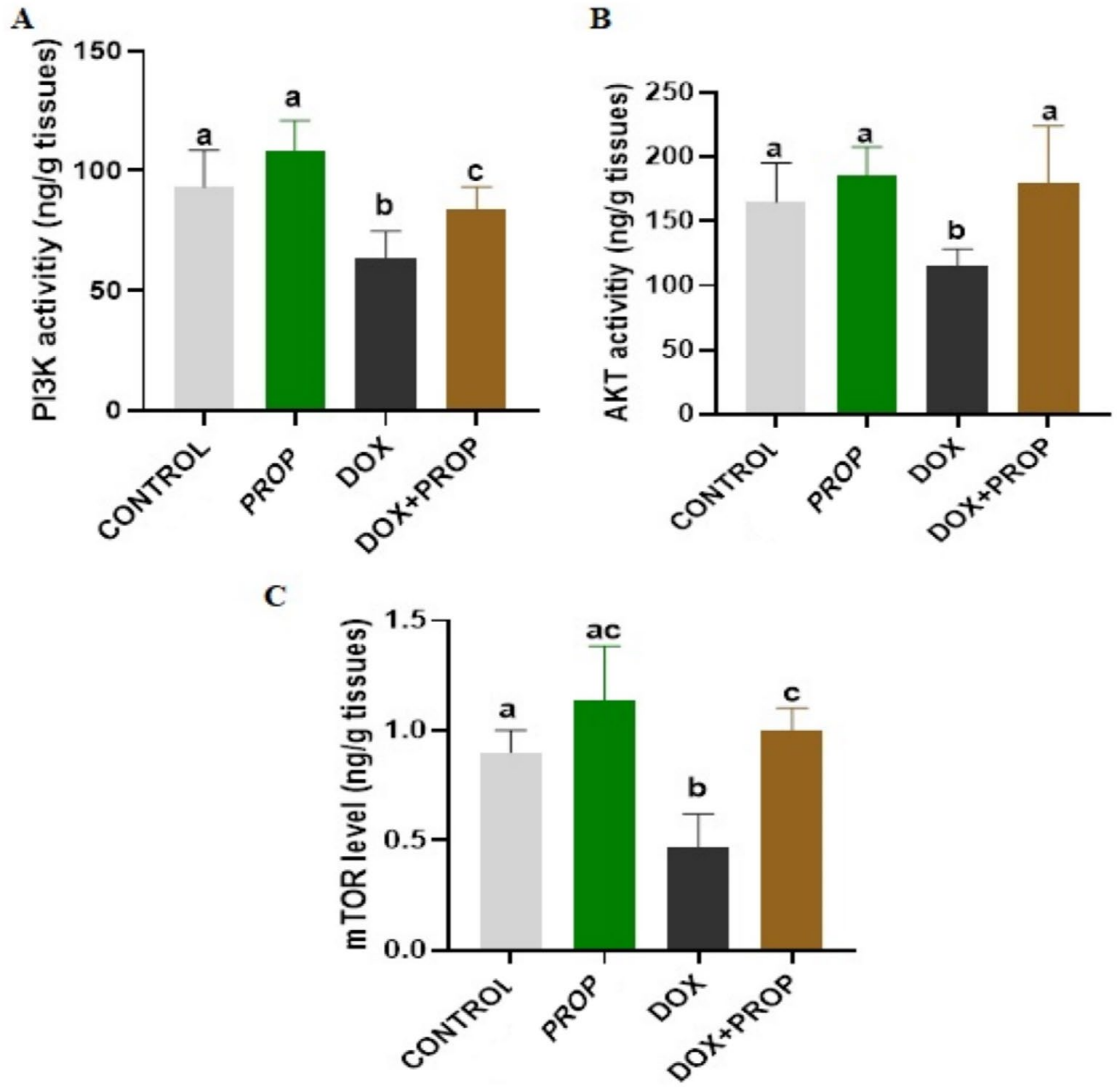
PI3K (A) and AKT activity (B) and mTOR levels (C) in brain tissue samples obtained from experimental groups. Different letters indicate statistical differences between groups (*p* < 0.05).

After spectrophotometric analysis, we determined that PI3K and AKT activity and mTOR levels were decreased in brain tissue samples obtained from the DOX group. However, we determined that the PROP application attenuated the DOX‐induced decrease in PI3K, mTOR, and AKT activity (Figure 1; *p* < 0.05).

### 3.3 Effects of PROP on Inflammation and Apoptosis Parameters in DOX‐Induced Brain Tissue

**FIGURE 2:**
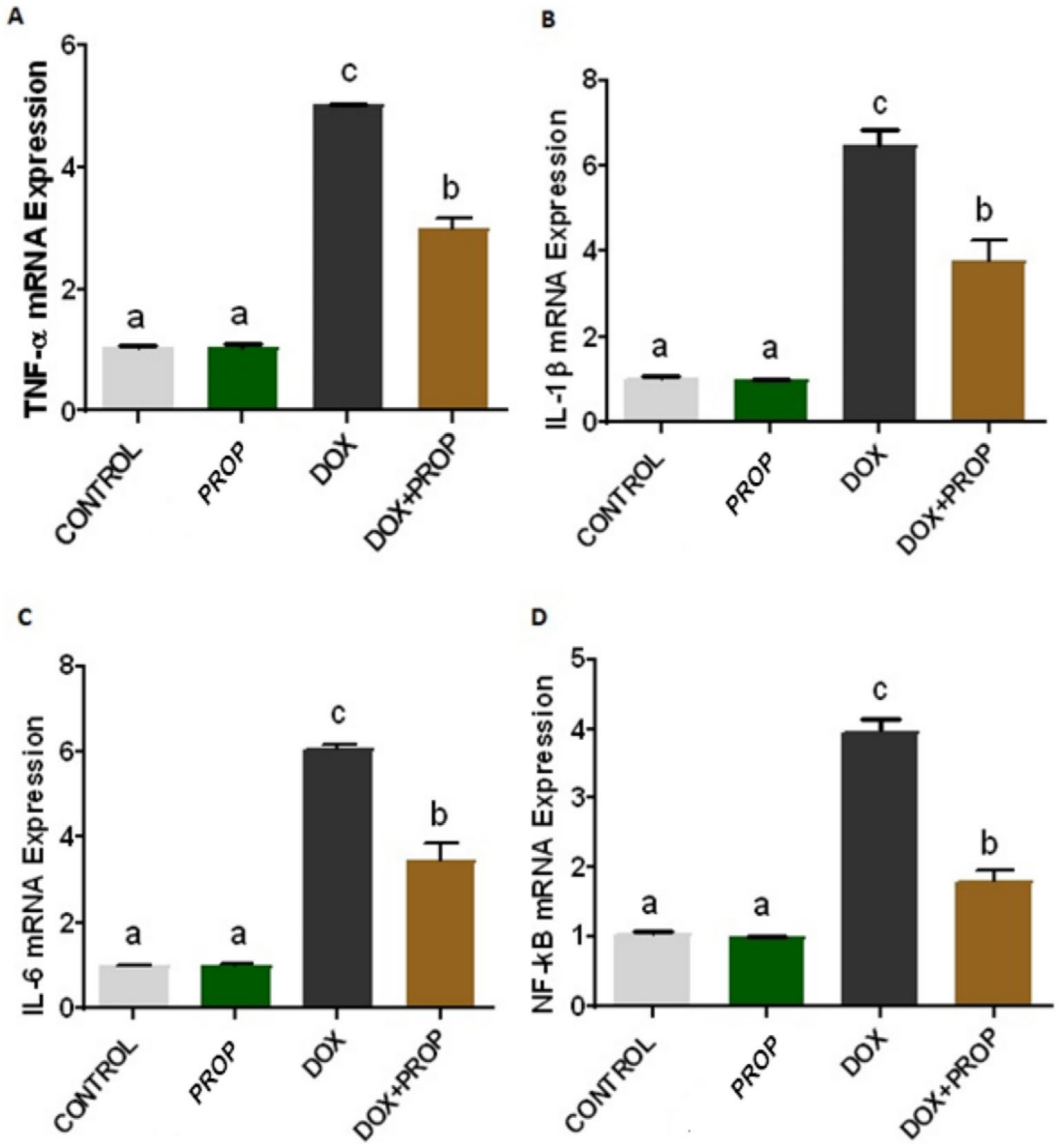
TNF‐α (A), IL‐1β (B), IL‐6 (C), and NF‐kB (D) mRNA expression levels in brain tissue samples obtained from experimental groups. Different letters indicate statistical differences between groups (*p* < 0.05).

**FIGURE 3:**
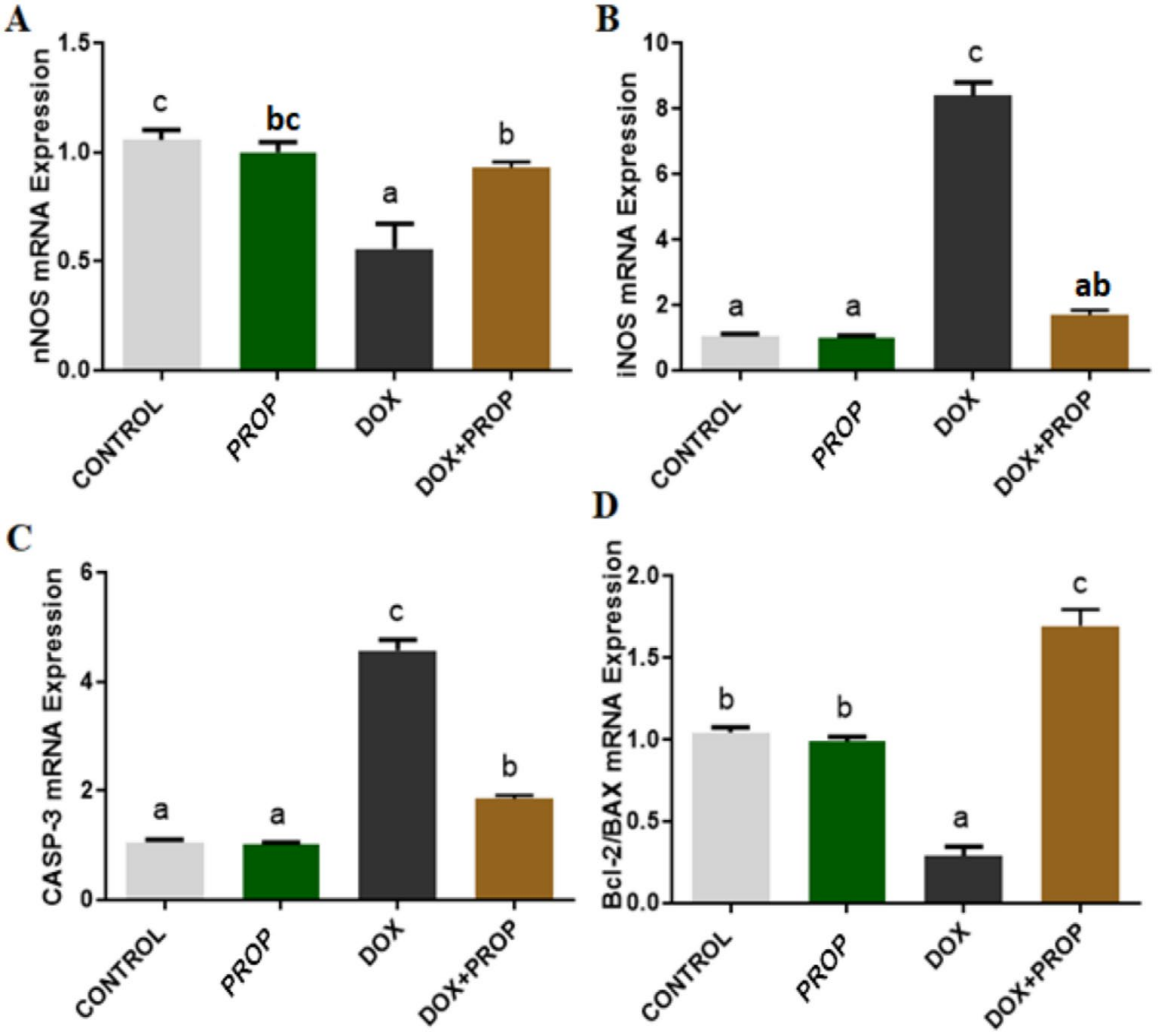
nNOS, iNOS, CASP‐3, and BCL2/BAX mRNA expression levels in brain tissue samples obtained from experimental groups. Different letters indicate statistical differences between groups (*p* < 0.05).

After real‐time PCR analyses, we determined that TNF‐α, IL‐1β, IL‐6, NF‐kB, iNOS, and CASP‐3 mRNA expression levels increased in brain tissue samples obtained from the DOX group. However, we determined that PROP application attenuated the DOX‐induced increase in TNF‐α, IL‐1β, IL‐6, NF‐kB, iNOS, and CASP‐3 mRNA expression levels (Figure 2; *p* < 0.05). On the other hand, we determined that nNOS and BCL2/BAX mRNA expression levels decreased in brain tissue samples obtained from the DOX group. However, we determined that PROP application attenuated the DOX‐induced decrease in nNOS and BCL2/BAX mRNA expression levels (Figure 3; *p* < 0.05).

### 3.4 Western Blot Analysis Results

**FIGURE 4:**
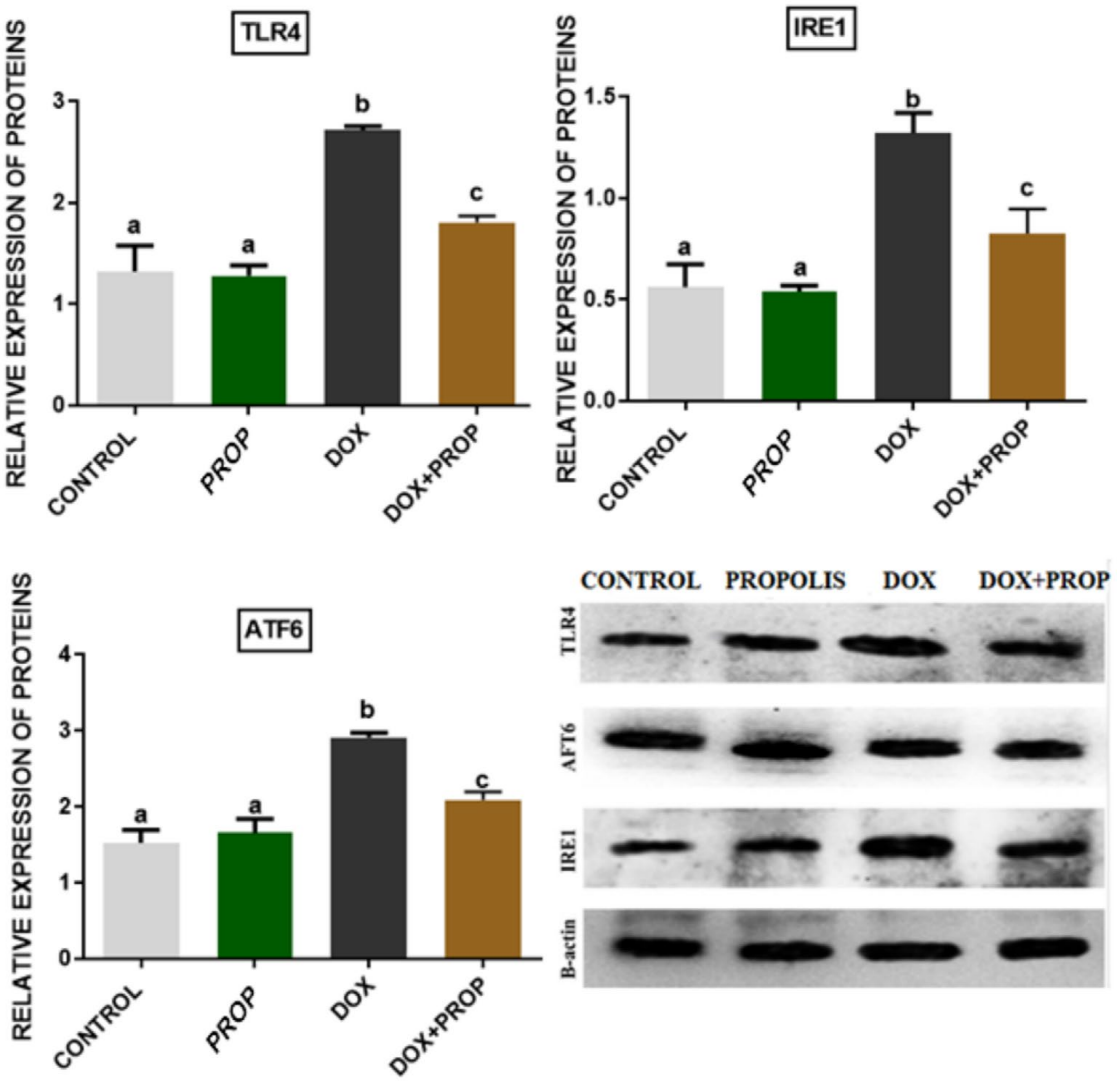
Relative protein expression of TLR4, IRE1, and ATF6 in brain tissue samples obtained from experimental groups. Different letters indicate statistical differences between groups (*p* < 0.05).

To evaluate the immune response and endoplasmic reticulum stress response in brain tissue samples obtained from experimental groups, TLR4, IRE1, and ATF6 protein expression was evaluated. We determined that TLR4, IRE1, and ATF6 protein expression levels increased in brain tissue samples obtained from the DOX group. However, we determined that PROP application attenuated the DOX‐induced increase in TLR4, IRE1, and ATF6 protein expression levels (Figure 4; *p* < 0.05).

### 3.5 Histopathological Findings

**FIGURE 5:**
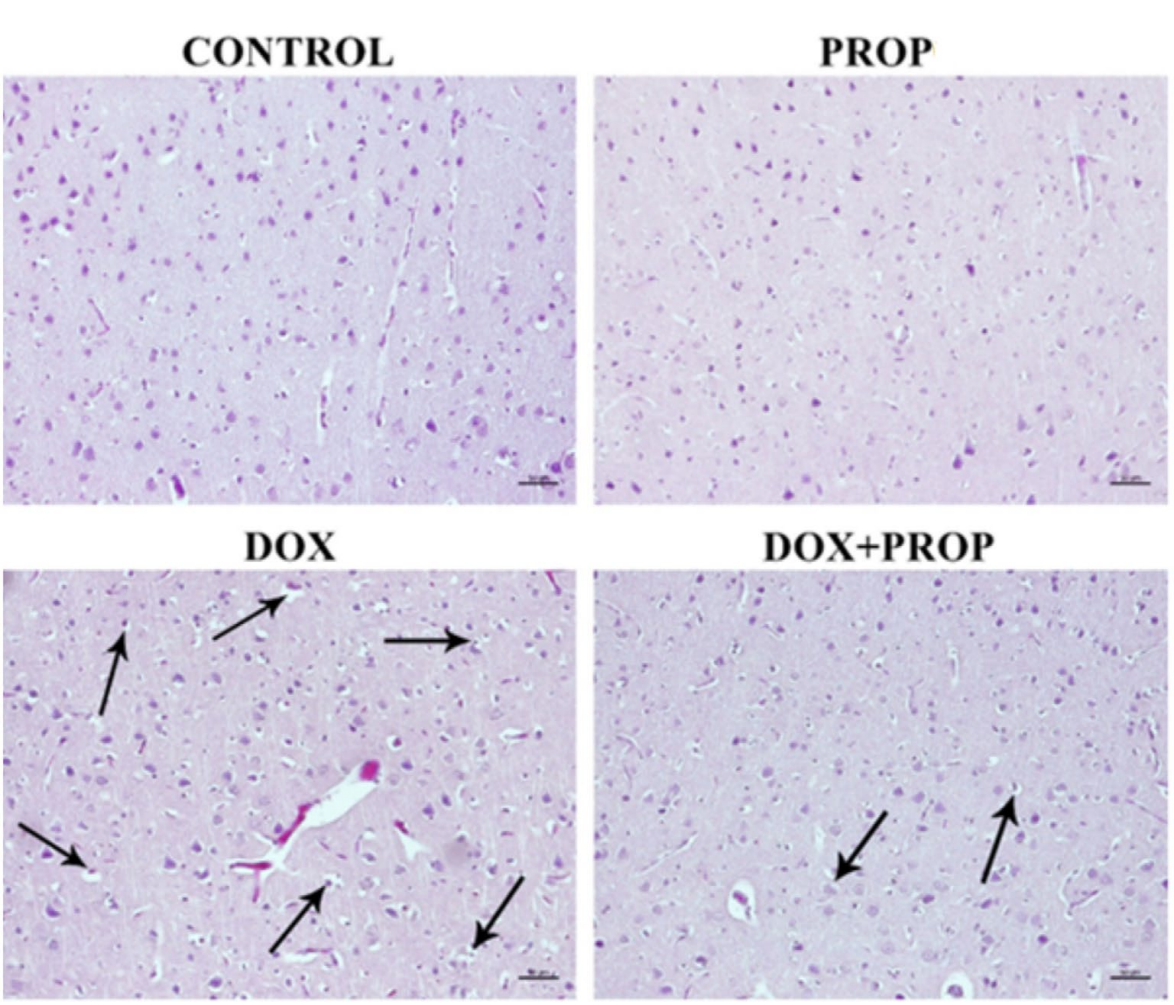
Rat brain tissue, frontal cortex histopathology, Crossman's Modified Mallory's Triple Stain, ×200 magnification, scale bar 50 μm. Arrow: Degenerated neurons and perineuronal vacuolization.

**FIGURE 6:**
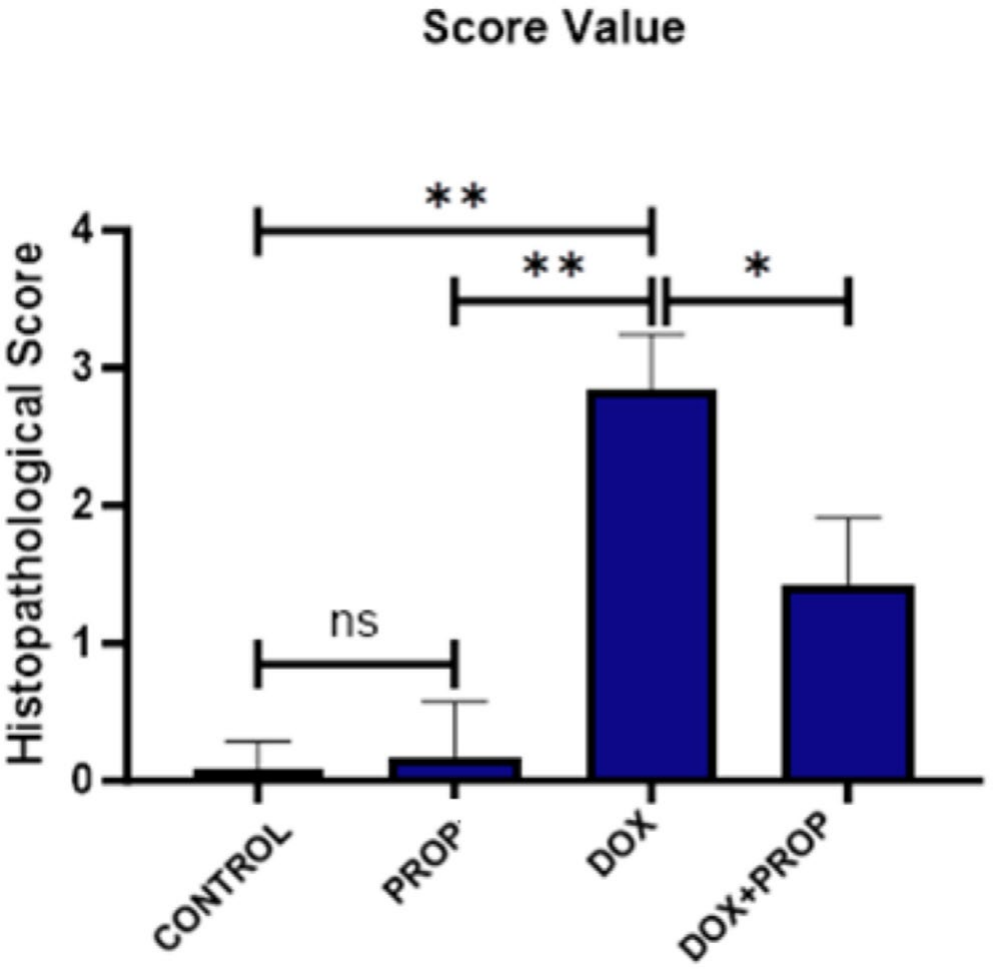
Evaluation of neuronal damage in the cerebral cortex. Values are given as mean ± SD (*n* = 7) and analyzed by one‐way ANOVA followed by Tukey's test. Asterisk indicates statistically significant differences between groups (*p < 0.05; **p < 0.001; ns: Insignificant).

No histopathological changes were detected in the control and PROP groups, and the brain tissues had a normal histological appearance. It was observed that most pyramidal neurons in the cerebral cortex areas in the DOX group were degenerated and vacuolated. In the DOX + PROP group, neuronal degeneration and vacuolation were significantly reduced and remained quite limited compared to the group exposed to DOX (Figure 5). It was determined that neuronal damage scores increased in the DOX group compared to the other groups. It was noted that the application of PROP together with DOX reduced neuronal damage scores. Sample images of all findings are presented in Figure 5, and the graph of histopathological scoring is presented in Figure 6.

### 3.6 Method Validation and Results Obtained for the Analysis of DOX in Brain Tissues Using UHPLC‐Orbitrap‐HRMS

**FIGURE 7:**
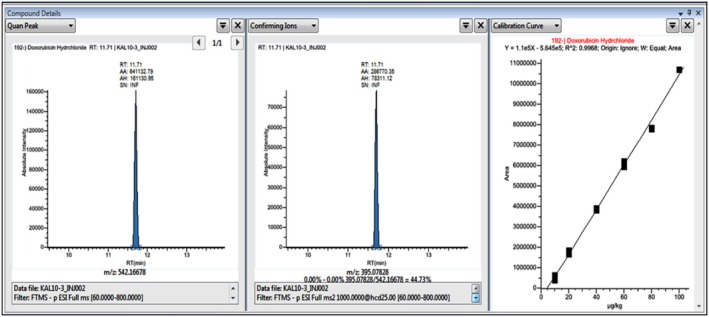
UHPLC‐Orbitrap‐HRMS Quan Peak (Parent Ion), Confirming Ion (m/z) (Fragment Ions) Chromatograms, and Matrix‐Affected Calibration Curve for DOX at a Concentration of 10 μg/kg.

**FIGURE 8:**
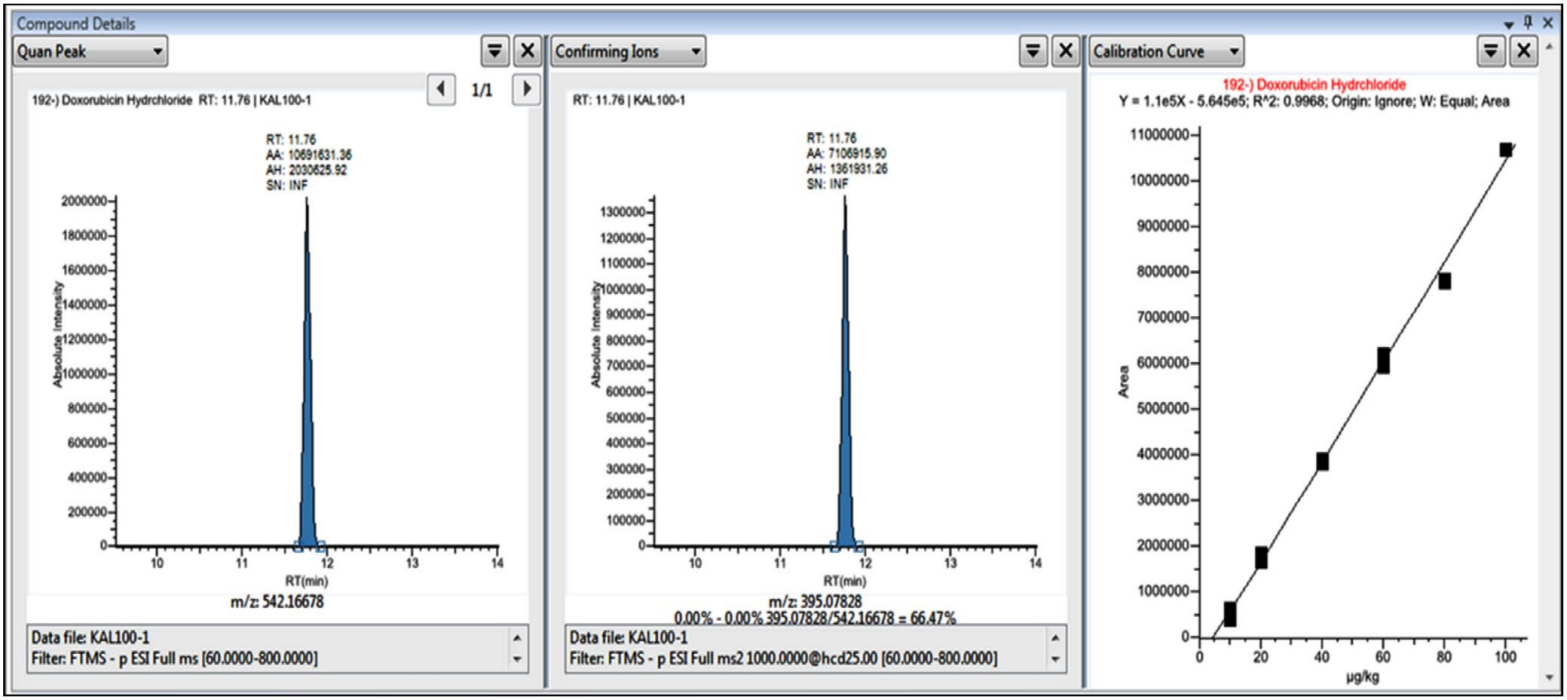
UHPLC‐Orbitrap‐HRMS Quan Peak (Parent Ion), Confirming Ion (m/z) (Fragment Ions) chromatograms and matrix effect calibration curve of DOX at a concentration of 100 μg/kg.

The analysis of DOX in samples was conducted using UHPLC‐Orbitrap‐HRMS. A DOX standard stock solution was prepared in methanol at 1000 μg/kg. Spikes were added to control samples without DOX to prepare matrix‐effect calibration for the analysis of DOX in brain tissue samples. The DOX standard was then diluted in 50% methanol‐50%, 0.5% acetic acid in an ultrapure water mixture to obtain 10, 20, 40, 60, 80, and 100 μg/kg concentrations. These prepared standard mixtures were analyzed using UHPLC‐Orbitrap‐HRMS to create matrix‐effect calibration curves. The Quan Peak (Parent Ion) and Fragment Ions (m/z) (Confirming Ion) chromatograms for 10 μg/kg were presented in matrix‐effect calibration Figure 7. In contrast, those for 100 μg/kg were shown in the matrix‐effect calibration 100 μg/kg Figure 8. Brain tissue samples were evaluated based on the prepared calibration graphs.

The Quan Peak (Parent Ion) and Confirming Ions (Fragment Ions) of DOX, retention time (RT), concentration ranges, determination coefficient (*R* ^2^) of the calibration curve, limit of detection (LOD) (μg/kg), limit of quantification (LOQ) (μg/kg), recovery, and recovery RSD (%) analytical parameters were determined by spiking six control samples without DOX with a concentration of 10 μg/kg. These parameters are presented in Table 5.

**TABLE 5:** Analytical parameters for the UHPLC‐Orbitrap‐HRMS analysis of DOX.

Compound	RT	Quan Peak (m/z)	Fragment Ions (m/z)	Linear range (ng/mL)	*R* ^2^	LOD (μg/kg) (*n* = 6)	LOQ (μg/kg) (*n* = 6)	% Recovery (*n* = 6)^a^	% Recovery RSD (*n* = 6)	ME^b^ (*n* = 6)	ME RSD
		Parent Ion (m/z)	Confirming Ion (m/z)								
DOX	11.76	542.16678	395.07858	10–100	0,9968	0.59	1.97	96.82	2.04	0,972	3.240

Abbreviations: LOD, limit of detection; LOQ, limit of quantification; ME, matrix effect; *R* ^2^, correlation coefficient.

^a^ Percent recovery was calculated by multiplying the ratio of the average peak area of the analyte added before extraction to the average peak area of the analyte added after extraction by 100 (*n* = 6).

^b^ The matrix effect was determined by calibration curves obtained from a matrix solution spiked with DOX into brain tissue and a brain tissue solution without DOX matrix.

According to the results of DOX analysis performed on brain tissues using UHPLC‐Orbitrap‐HRMS; in the analysis where 10 μg/kg DOX concentration (Control +10 μg/kg DOX spike) was spiked to the control group for the accuracy and reliability of the analysis, 9.72 μg/kg DOX was detected and 97.2% recovery was achieved. In contrast, DOX was not detected in the control group or the other groups.

### 3.7 Method Validation and Results for the Analysis of Phytochemicals in PROP Extract by UHPLC‐Orbitrap‐HRMS

**FIGURE 9:**
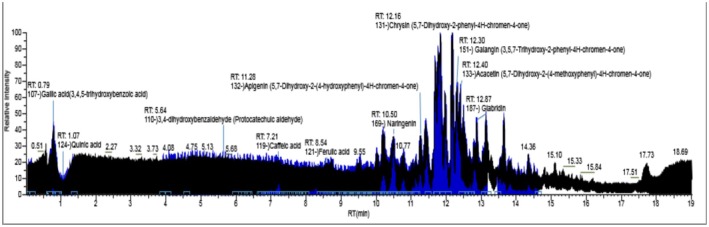
TIC profile of phytochemical standard compounds of PROP extract.

Method validations were conducted for UHPLC‐Orbitrap‐HRMS analyses to determine the 70 phytochemical profiles in the PROP extract. For the phytochemical compounds to be analyzed, retention time (RT), Quan Peak (m/z), ion mode (polarity), Confirming Ions (m/z), the correlation coefficient of calibration curves (*R* ^2^), and linear range (concentration ranges) (μg/L) were determined. One of the prepared PROP extracts was selected, and the analytes to be analyzed were diluted to a non‐signal level in the extract prepared by diluting the extract. Standard concentrations were prepared by adding 3 standard additions to determine the limit of detection (LOD) (μg/L) and limit of quantification (LOQ) (μg/L). The recovery percentage and recovery percentage RSD value were determined by spiking the sample at concentrations of 10 μg/L, and the analytical parameters were calculated and presented in Table 6. The TIC profile of phytochemical standard compounds in the PROP extract is shown in Figure 9.

**TABLE 6:** Analytical parameters for UHPLC‐Orbitrap‐HRMS analysis of phytochemicals and identification of phytochemicals in PROP extract.

No	Target compounds	RT	Parent Ion (m/z)	Fragment Ions (m/z)	Ion mode	*R* ^2^	Liner range (μg/kg)	10 ng/mL (*n* = 3)				μg/g PROP extract
			Quan Peak (m/z)	Confirming Ions (m/z)				LOD (μg/kg)	LOQ (μg/kg)	% Recovery	% Recover RSD	
**1**	**Benzoic acid**	**8.93**	**121.02950**	**121.02950**	**—**	**0.9913**	**10–1000**	**0.70**	**2.33**	**103.80**	**2.24**	**990.17**
**2**	**4‐Hydroxybenzoic acid**	**6.15**	**137.02442**	**93.03471**	**—**	**0.9921**	**10–100**	**1.92**	**6.39**	**112.48**	**5.68**	**60.73**
3	Salicylic acid	9.9	137.02442	93.03468	—	0.9946	10–100	1.85	6.17	111.69	5.52	N.D.
4	3‐hydroxybenzoic acid	6.93	137.02442	93.03471	—	0.9926	10–100	1.55	5.15	109.45	4.71	N.D.
5	3‐hydroxyphenylacetic acid	6.79	151.04007	107.05045	—	0.9923	10–100	2.38	7.93	106.71	7.43	N.D.
6	Syringic acid	7.48	197.04555	197.04555	—	0.9949	10–100	1.53	5.09	108.09	4.71	N.D.
**7**	**Gallic acid**	**0.79**	**169.01425**	**125.02461**	**—**	**0.9903**	**10–100**	**2.32**	**7.73**	**112.73**	**6.86**	**29.361**
8	Protocatechuic acid	4.26	153.01933	109.02949	—	0.9908	10–100	2.46	8.19	83.59	9.80	N.D.
9	Protocatechuic acid ethyl ester	9.14	181.05063	108.02187	—	0.9905	10–100	4.58	15.26	70.58	21.62	N.D.
**10**	**3,4‐dihydroxybenzaldehyde**	**5.64**	**137.02442**	**136.01671**	**—**	**0.9909**	**10–100**	**3.54**	**11.80**	**76.00**	**15.53**	**390.85**
11	2.4‐dihydroxybenzoic acid	7.12	153.01933	67.01888	—	0.9935	10–100	1.48	4.92	109.00	4.51	N.D.
**12**	**Vanillic acid**	**7.09**	**167.03498**	**108.02173**	**—**	**0.9938**	**10–100**	**1.82**	**6.07**	**111.39**	**5.45**	**65.3**
**13**	**Vanillin**	**7.6**	**151.04007**	**108.02178**	**—**	**0.9913**	**10–100**	**2.16**	**7.21**	**108.27**	**6.66**	**235.9**
14	Gentisic acid	6.45	153.01933	153.01933	—	0.9913	10–100	0.66	2.22	103.06	2.15	N.D.
**15**	**Trans Cinnamic acid**	**10.28**	**147.04515**	**147.04515**	**—**	**0.9952**	**10–100**	**1.82**	**6.06**	**102.10**	**5.94**	**435.91**
**16**	**p‐Coumaric acid**	**8.26**	**163.04007**	**119.05027**	**—**	**0.9928**	**10–1000**	**1.12**	**3.73**	**98.96**	**3.77**	**1612.25**
**17**	**Caffeic acid**	**7.21**	**179.03498**	**135.04509**	**—**	**0.9917**	**10–1000**	**1.16**	**3.88**	**107.04**	**3.62**	**3625.28**
**18**	**Caffeic acid phenhyl ester**	**11.99**	**283.09758**	**135.04526**	**—**	**0.9980**	**10–1000**	**1.94**	**6.46**	**101.78**	**6.34**	**2224.27**
**19**	**Ferulic acid**	**8.52**	**193.05063**	**134.03751**	**—**	**0.9933**	**10–1000**	**0.87**	**2.89**	**101.60**	**2.84**	**466.1**
**20**	**Sinapic acid**	**8.54**	**223.06120**	**193.01436**	**—**	**0.9934**	**10–100**	**1.32**	**4.39**	**105.72**	**4.15**	**17.17**
**21**	**Chlorogenic acid**	**7.03**	**353.08781**	**191.05624**	**—**	**0.9976**	**10–100**	**0.97**	**3.23**	**104.04**	**3.11**	**25.54**
**22**	**Quinic acid**	**1.24**	**191.05611**	**85.02962**	**—**	**0.9939**	**10–100**	**0.95**	**3.17**	**104.01**	**3.05**	**96.23**
**23**	**3‐(4‐Hydroxyphenyl) PROPionic acid**	**7.6**	**165.05572**	**108.02195**	**—**	**0.9927**	**10–100**	**2.35**	**7.83**	**95.49**	**8.20**	**54.91**
24	α‐Cyano‐4‐hydroxycinnamic acid	9.26	188.03532	93.03470	—	0.9925	10–100	0.67	2.23	101.56	2.20	N.D.
25	Catechin	6.38	289.07176	109.02975	—	0.9943	10–100	1.40	4.65	108.28	4.30	N.D.
**26**	**Chrysin**	**12.18**	**253.05063**	**253.05063**	**—**	**0.9907**	**10–1000**	**0.97**	**3.23**	**105.36**	**3.07**	**4830.50**
**27**	**Apigenin**	**11.28**	**269.04555**	**117.03464**	**—**	**0.9932**	**10–1000**	**1.42**	**4.72**	**105.45**	**4.48**	**1421.03**
**28**	**Acacetin**	**12.39**	**283.06120**	**268.03717**	**—**	**0.9911**	**10–100**	**2.48**	**8.27**	**111.99**	**7.39**	**328.24**
29	Vicenin 2	7.83	593.15119	473.10941	—	0.9945	10–100	2.02	6.73	111.78	6.02	N.D.
30	Apigenin 7‐glucuronide	10.37	445.07763	269.04535	—	0.9923	10–100	1.36	4.52	103.63	4.37	N.D.
31	Apigenin 7‐glucoside	9.57	431.09837	269.04544	—	0.9947	10–100	1.45	4.83	108.28	4.46	N.D.
**32**	**Genkwanin**	**12.39**	**283.06120**	**268.03745**	**—**	**0.9911**	**10–100**	**0.67**	**2.23**	**102.66**	**2.18**	**320.43**
33	Apiin	9.44	563.14063	269.04498	—	0.9923	10–100	2.02	6.72	111.75	6.01	N.D.
34	Vitexin	8.69	431.09837	311.05603	—	0.9939	10–100	1.15	3.83	106.57	3.60	N.D.
35	Schaftoside	8.33	563.14063	443.09949	—	0.9908	10–100	1.81	6.05	105.55	5.73	N.D.
**36**	**Rutin**	**9.23**	**609.14611**	**300.02777**	**—**	**0.9912**	**10–100**	**1.40**	**4.67**	**100.49**	**4.65**	**29.12**
**37**	**Luteolin**	**10.75**	**285.04046**	**175.04039**	**—**	**0.9932**	**10–1000**	**1.35**	**4.52**	**97.87**	**4.61**	**821.24**
38	Luteolin‐7‐O‐glucuronide	9.76	461.07255	285.04056	—	0.9938	10–100	0.74	2.45	96.90	2.53	N.D.
**39**	**Diosmetin**	**11.32**	**299.05611**	**122.90711**	**—**	**0.9954**	**10–1000**	**1.99**	**6.62**	**112.49**	**5.88**	**1200.8**
40	Luteoloside	10.39	447.09328	285.04059	—	0.9946	10–100	1.20	3.99	101.24	3.94	N.D.
41	Luteolin 7‐rutinoside	8.99	593.15119	285.04065	—	0.9923	10–100	0.83	2.76	103.99	2.65	N.D.
**42**	**Galangin**	**12.32**	**269.04555**	**269.04555**	**—**	**0.9936**	**10–1000**	**0.98**	**3.27**	**96.05**	**3.41**	**2500.91**
**43**	**Isoquercitrin**	**9.23**	**463.08820**	**300.02768**	**—**	**0.9939**	**10–100**	**0.75**	**2.49**	**104.33**	**2.39**	**3.6**
**44**	**Quercetin**	**10.47**	**301.03538**	**151.00342**	**—**	**0,9900**	**10–1000**	**0.73**	**2.42**	**97.04**	**2.49**	**1182.84**
**45**	**Narcissin**	**9.8**	**623.16176**	**315.05139**	**—**	**0.9918**	**10–100**	**1.36**	**4.53**	**99.85**	**4.54**	**16.5**
46	Quercetin 3‐rutinoside 7‐glucoside	7.43	287.05536	135.04512	—	0.9934	10–100	0.23	0.77	98.86	0.78	N.D.
**47**	**Isorhamnetin**	**11.25**	**315.05103**	**300.02780**	**—**	**0.9958**	**10–100**	**0.54**	**1.80**	**97.64**	**1.84**	**875.4**
**48**	**Kaempferol**	**9.23**	**285.04046**	**136.01686**	**—**	**0.9924**	**10–100**	**0.58**	**1.94**	**97.21**	**1.99**	**4.4**
49	Afzelin	10.27	431.09837	285.04028	—	0.9951	10–100	1.43	4.77	97.78	4.88	N.D.
50	Kaempferide	10.28	299.05611	284.03265	—	0.9973	10–100	0.75	2.49	95.74	2.60	N.D.
**51**	**Nicotiflorin**	**9.73**	**593.15119**	**285.04041**	**—**	**0.9915**	**10–100**	**0.90**	**3.00**	**96.67**	**3.10**	**7.32**
**52**	**Astragalin**	**9.76**	**447.09328**	**284.03281**	**—**	**0.9936**	**10–100**	**1.58**	**5.28**	**99.41**	**5.31**	**< LOQ**
**53**	**Leucoside**	**9.8**	**287.05536**	**151.00372**	**—**	**0.9934**	**10–100**	**0.52**	**1.73**	**100.70**	**1.72**	**174.6**
**54**	**Fisetin hydrate**	**9.88**	**285.04046**	**135.00900**	**—**	**0.9913**	**10–80**	**0.97**	**3.24**	**104.76**	**3.09**	**15.52**
**55**	**Naringenin**	**10.50**	**271.06120**	**119.05035**	**—**	**0.9946**	**10–1000**	**1.01**	**3.38**	**104.16**	**3.25**	**4310.7**
**56**	**Sakuranetin**	**11.7**	**285.07685**	**119.05032**	**—**	**0.9921**	**10–100**	**1.28**	**4.27**	**104.16**	**4.10**	**209.11**
57	Narirutin	8.83	579.17193	271.06107	—	0.9926	10–100	1.39	4.63	103.35	4.48	N.D.
58	Liquiritigenin	9.85	255.06628	255.06592	—	0.9943	10–100	0.69	2.30	96.84	2.37	N.D.
59	Daidzin	8.05	415.10346	253.05191	—	0.9947	10–100	1.09	3.62	103.48	3.50	N.D.
**60**	**Formononetin**	**11.38**	**267.06628**	**252.04263**	**—**	**0.9917**	**10–100**	**0.94**	**3.14**	**95.10**	**3.30**	**14.9**
**61**	**Ellagic acid**	**9.43**	**300.99899**	**300.99872**	**—**	**0.9943**	**10–100**	**1.11**	**3.71**	**106.75**	**3.47**	**75.8**
**62**	**Esculin hydrate**	**6.24**	**339.07216**	**177.01930**	**—**	**0.9960**	**10–100**	**1.18**	**3.94**	**104.58**	**3.77**	**6.9**
63	Phloridzin	9.36	435.12967	273.07648	—	0.9924	10–100	1.64	5.48	108.91	5.03	N.D.
64	Rosmarinic acid	9.52	359.07724	161.02451	—	0.9935	10–100	0.82	2.75	104.66	2.62	N.D.
**65**	**Glabridin**	**12.88**	**323.12888**	**135.04533**	**—**	**0.9967**	**10–100**	**0.90**	**3.00**	**102.28**	**2.94**	**10.93**
66	Arbutin	0.79	271.08233	108.02180	—	0.9904	10–100	1.22	4.06	99.29	4.08	N.D.
67	Emodin	13.44	269.04555	225.05573	—	0.9909	10–100	0.70	2.34	97.21	2.40	N.D.
68	Etoposide	9.89	611.17351	405.09308	—	0.9906	10–100	1.49	4.98	97.86	5.09	N.D.
69	DOX Hydrchloride	11.76	542.16678	395.07828	—	0.9929	10–100	2.16	7.18	92.67	7.75	N.D.
70	Ethylgallate	7.83	197.04555	124.01682	—	0.9920	10–100	0.72	2.39	96.27	2.49	N.D.

Abbreviations: Confirming Ions, fragment ions (m/z) of phytochemical compound standards; LOD, limit of detection; LOQ, limit of quantification; N.D., not detected; Quan Peak, parent Ion (m/z) of phytochemical compound standards; *R* ^2^, correlation coefficien; RSD, relative standard deviation; RT, retention time.

A total of 38 phytochemicals were identified, including benzoic acid, 4‐hydroxybenzoic acid, gallic acid, 3,4‐dihydroxybenzaldehyde, vanillic acid, vanillin, trans‐cinnamic acid, coumaric acid, caffeic acid, caffeic acid phenyl ester, ferulic acid, sinapic acid, chlorogenic acid, quinic acid, 3‐(4‐hydroxyphenyl) PROPionic acid, chrysin, apigenin, acacetin, genkwanin, rutin, luteolin, diosmetin, galangin, isoquercitrin, quercetin, narcissin, isorhamnetin, kaempferol, nicotiflorin, astragalin, leucoside, fisetin hydrate, naringenin, sakuranetin, formononetin, ellagic acid, esculin hydrate, and glabridin. Among these, chrysin was found at 4830.50 μg/g, naringenin at 4310.7 μg/g, caffeic acid at 3625.28 μg/g, galangin at 2500.91 μg/g, caffeic acid phenyl ester at 2224.27 μg/g, coumaric acid at 1612.25 μg/g, apigenin at 1421.03 μg/g, diosmetin at 1200.8 μg/g, quercetin at 1182.84 μg/g, benzoic acid at 990.17 μg/g, isorhamnetin at 875.4 μg/g, and luteolin at 821.24 μg/g, indicating these phytochemicals as dominant.

## 4 Discussion

With their cytotoxic actions, the majority of medications used in cancer treatment stop malignant cells from growing and proliferating and instead induce their death (Abd El‐Gany et al. 2016). DOX, a member of the anthracycline group of chemotherapeutic agents, is useful in the treatment of cancer but has several negative consequences (Arivalagan et al. 2018). Sarcoma, lymphoma, prostate cancer, thyroid, lung, and breast carcinomas are among the hematological malignancies for which it is most commonly used (Abd El‐Gany et al. 2016). The primary worry when using chemotherapy medications is their potential for toxicity. Numerous chemotherapeutic medications have been linked to neurotoxicity, according to studies. One way that DOX may harm brain tissue is significant lipid peroxidation in the heart, kidney, liver, ovary, and testicular tissues (Kiyomiya et al. 2001).

It is accepted that DOX directly or indirectly alters the gene expression of different enzymes at transcriptional or translational stages through the production of free radicals, which leads to changes in the activity of antioxidant enzymes (Denard et al. 2011; Kara et al. 2016). According to some studies, nitric oxide (NO), superoxide, and hydroxyl radicals are the free radicals responsible for pathogenesis. In addition, antioxidant enzymes such as superoxide dismutase (SOD) and catalase (CAT) are reduced by lipid peroxidation products such as MDA, which are produced by free radicals and damage cells (Gündoğdu et al. 2023; Karaman et al. 2006; Hirai et al. 2006). Antioxidant treatment trials have attracted attention due to studies showing the role of free radicals and antioxidant enzymes in the pathophysiology of DOX‐associated toxicity (Tulubas et al. 2015). Oxidative stress is one of the main mechanisms of DOX‐induced neurotoxicity. In our study, it was determined that PROP strengthens antioxidant defense by increasing SOD and CAT levels, reduces lipid peroxidation by decreasing MDA levels, and protects cellular redox balance by increasing GSH levels. These findings parallel the study conducted by Elbaz et al. (2010). In the said study, PROP was shown to suppress oxidative stress in brain tissue and increase antioxidant enzyme activity. Similarly, Wang et al. (2020) reported that PROP reduces oxidative stress in neurodegenerative diseases. In addition, da Silva et al. (2018) stated that the antioxidant capacity of PROP increases thanks to its flavonoid components and that these components have a protective effect against oxidative damage. In addition, the regulation in nNOS and iNOS levels suggests that PROP has a neuroprotective effect by supporting nitric oxide homeostasis. This was also stated in the study by Kitamura (2019), and it was stated that PROP protects cell health by reducing neuronal oxidative stress.

Numerous chronic diseases, including cancer, diabetes, heart disease, and neurological disorders, are linked to inflammation. On the other hand, oxidative stress has been documented to activate various transcription factors, including p53, NFkB, and AP‐1 (Karamese et al. 2016). By increasing the levels of pro‐inflammatory cytokines and decreasing the levels of anti‐inflammatory cytokines, DOX causes inflammation (Ujah et al. 2021). DOX administration triggered the inflammatory response, increasing the levels of NFκB, TLR4, IL‐6, IL‐1β, and TNF‐α. However, PROP extract reduced inflammation by suppressing these parameters. In the literature, Zulhendri et al. (2022) showed that PROP reduced the production of inflammatory cytokines by inhibiting the NfκB and TLR4 signaling pathways. Furthermore, Oršolić and Jazvinšćak Jembrek (2022) reported that PROP suppressed inflammation in brain tissue by decreasing the levels of IL‐6 and TNF‐α. Our study is consistent with these results and supports the idea that PROP has a neuroprotective effect by inhibiting the activity of NfκB, a central regulator of inflammation. In addition, Nattagh‐Eshtivani et al. (2022) reported that PROP suppresses inflammatory processes by reducing IL‐1β and TNF‐α levels and inhibits microglial activation.

ER is an organelle with a high threshold to both intracellular and external stimuli (Flessa et al. 2022). p‐PERK, IRE1α, and ATF6 are three transmembrane proteins that assemble unfolded proteins and dissociate from GRP78 when ER stress is induced for any reason, leading to an increase in the amount of unfolded or misfolded proteins in the cell (Kara et al. 2023). ER stress is characterized by an increase in the expression levels of GRP78, CHOP, ATF6, p‐IRE1, sXBP1, and p‐PERK. In our study, DOX application triggered ER stress by increasing ATF6 and p‐IRE1 activity. PROP administration was observed to suppress these parameters. Similarly, Scorza et al. (2024) reported that PROP maintains cellular homeostasis by inhibiting ER stress pathways. In addition, Chen et al. (2023) showed that PROP maintains cellular protein balance by reducing ER stress and reducing apoptotic cell death.

The majority of the pyramidal neurons in the DOX group's cerebral cortex regions were shown to have deteriorated in our histological analyses, along with signs of brain injury such as vacuolation. According to animal studies, DOX application results in cortical injury, which is in line with our findings (Kosoko et al. 2017). Furthermore, our results demonstrate that PROP extract mitigates the histologically induced brain damage caused by DOX. According to several recent studies, ROS are necessary to initiate the apoptotic process of the cell (Gelen et al. 2023; Gür et al. 2022; Sengul et al. 2023). Caspase‐3 and Bcl‐2 are two members of the Bcl‐2/Bax protein family, which initiate a pathway in which apoptotic mechanisms occur (Gelen et al. 2021; Chen et al. 2019). Ujah et al. (2021) found that when rats were exposed to DOX‐induced testicular toxicity, the expression of Bcl‐2 decreased, and the expression of Caspase‐3 increased in the toxicity group. In our study, DOX induced apoptosis by increasing Bax levels and decreasing Bcl‐2 levels. However, PROP reversed these ratios and suppressed the apoptotic process. Similar findings were reported in the study by Abdel‐Rahman et al. (2020), which showed that PROP reduced apoptotic cell death in brain tissue. In addition, activation of the PI3K/Akt/mTOR pathway was identified as one of the mechanisms by which PROP protects cell health. Forma and Bryś (2021) indicated that PROP affects the PI3K/Akt pathway. Additionally, Chen et al. (2022) showed that PROP controls autophagy by regulating mTOR signaling in neurons, thereby modulating cellular stress responses.

PROP contains numerous phenolic compounds that have positive effects on human health. Phenolic compounds also prevent oxidation and enhance the chemical stability of food products (Koksal and Gulcin 2008). Most of the biological effects of PROP are provided by flavonoids and other polyphenols; this group includes flavones, flavonols, flavanones, and dihydroflavonols (Guzelmeric et al. 2021). Among the components of PROP are benzoic acid, gallic acid, 4‐hydroxybenzoic acid, p‐coumaric acid, chlorogenic acid, quinic acid, caffeic acid, caffeic acid phenethyl ester, gallic acid, chrysin, apigenin, acacetin, rutin, luteolin, galangin, quercetin, nicotiflorin, astragalin, naringenin, kaempferol, rutin, quercetin, naringenin, and isoquercitrin. These components enhance the effectiveness of the digestive system, antioxidant capacity, and metabolic, physiological, and immune functions of body tissues (Apak et al. 2022). The phytochemicals identified in PROP, as mentioned in the literature, were found to be consistent with the phytochemicals detected in the PROP extract used in the study. Among the components of PROP, more than 300 compounds have been identified in various types of PROP, including phenolic acids and esters, flavonoids, triterpenes, alcohols, aromatic aldehydes, fatty acids, stilbenes, steroids, lignans, amino acids, and sugars (Oleggrio et al. 2019). In the literature, there are numerous studies on the chemical profile of natural products using UHPLC‐Orbitrap‐HRMS (Dursun et al. 2025; Ceylan et al. 2024). In this context, this report is an important study in terms of determining the chemical profile of PROP and the impact of the phytochemicals it contains. Phenolic compounds found in natural resources are highly important for human nutrition. Due to their biological effects, such as antioxidant Properties, they have attracted significant interest (Koksal and Gulcin 2008).

## 5 Conclusion

The results of this study suggest that DOX treatment causes the production of ROS, which in turn stimulates ROS‐mediated direct apoptosis. Moreover, ROS‐induced endoplasmic reticulum stress results in the suppression of mitochondrial BCL2 and increased production of Bax, which in turn stimulates the formation of caspase‐3, which causes apoptosis. Once more, elevating ROS causes endoplasmic reticulum stress, which in turn causes NF‐kB to develop in the nucleus. This, in turn, raises cytokine levels, promoting apoptosis. Nevertheless, we discovered that the administration of PROP extract inhibited these DOX‐stimulated signaling pathways. These results suggested that PROP extract may be an adjuvant therapy to lessen the adverse effects of DOX‐dependent oxidative stress, ER stress, inflammation, and apoptotic effects on brain tissue.

## Author Contributions

Research and technique. And writing innovative validation software: V.G., M.B., I.Ç., İ.D., and A.K. Writing: V.G., M.B., I.Ç., İ.D., and A.K. Preparing the initial draft. Conceptualization: Every author has reviewed and approved the published version of the study.

## Conflicts of Interest

The authors declare no conflicts of interest.

## Data Availability

The article or its supplemental materials include data.
